# Cold-hot nature identification of Chinese herbal medicines based on the similarity of HPLC fingerprints

**DOI:** 10.3389/fchem.2022.1002062

**Published:** 2022-09-20

**Authors:** Guohui Wei, Ronghao Jia, Zhiyong Kong, Chengjie Ji, Zhenguo Wang

**Affiliations:** ^1^ Key Laboratory of Theory of TCM, Ministry of Education of China, Shandong University of Traditional Chinese Medicine, Jinan, China; ^2^ College of Intelligence and Information Engineering, Shandong University of Traditional Chinese Medicine, Jinan, China

**Keywords:** Chinese herbal medicines, nature identification, HPLC, similarity metric, cold-hot nature

## Abstract

The nature theory of Chinese herbal medicines (CHMs) is the core theory of traditional Chinese medicine (TCM). Cold-hot nature is an important part of CHM nature. It is found that the material basis of cold-hot nature is CHM ingredients. To test the scientific hypothesis that “CHMs with similar cold-hot nature should have similar material basis,” we explored an intelligent method for cold-hot nature identification of CHMs based on the feature similarity of CHM ingredients in this work. Sixty one CHMs were selected for cold-hot nature identification. High performance liquid chromatography (HPLC) was used to separate the chemical ingredients of CHMs and extract the feature information of CHM ingredients. A distance metric learning algorithm was then learned to measure the similarity of HPLC fingerprints. With the learned distance metric, cold-hot nature identification scheme (CHNIS) was proposed to build an identification model to evaluate the cold-hot nature of CHMs. A number of experiments were designed to verify the effectiveness and feasibility of the proposed CHNIS model. The total identification accuracy rate of 61 CHMs is 80.3%. The performance of the proposed CHNIS algorithm outperformed that of the compared classical algorithms. The experimental results confirmed our inference that CHMs with similar cold-hot nature had similar composition of substances. The CHNIS model was proved to be effective and feasible.

## 1 Introduction

The nature theory of Chinese herbal medicines (CHMs) is one of the core contents of traditional Chinese medicine (TCM), which has been concerned by scholars and research institutions for many years. There are four types as for the nature of CHMs: Cool, hot, and warm, which can be summarized as cold and hot nature (Ouyang et al., 2006; Gao and Chen, 2007). Especially, according to this theory, CHMs are used as drugs to treat diseases. Hot nature medicines are used to treat cold syndrome, and cold nature medicines are used to treat hot syndrome. The cold-hot nature theory has become an important principle for TCM treatment (Fu et al., 2017). Therefore, correctly identifying the nature of CHMs is crucial in TCM research.

The field of studying cold-hot nature of CHMs has attracted much attention, and yielded a lot of results. Some studies found that cold-hot nature of CHMs was closely related to energy metabolism, including ATPase activity and oxygen consumption (Mi et al., 2021; Su et al., 2022). CHMs with cold nature can significantly reduce the level of energy metabolism in normal rats (Mi et al., 2021), and CHMs with hot nature may regulate the energy metabolism of skeletal muscle by promoting the decomposition of muscle glycogen and increasing the activity of SDH enzyme, so as to produce more ATP (Huang et al., 2010). Some studies analyzed the cold-hot nature of CHMs according to their material basis (Fu et al., 2017; Wei et al., 2019a). Scientists proposed various scientific hypotheses to prove that the material basis of cold-hot nature was the composition of CHMs. Our group put forward the scientific hypothesis of “tri-element of property-effect-material” and carried out a lot of experimental work to verify it (Zhang, 2012). Many researches have demonstrated that cold-hot nature of CHMs is based on substance (Wei et al., 2019a). Xue’s group explored to extract the ingredient information of CHMs with chemical fingerprints, and build an identification model for nature prediction (Zhang, 2012). Some studies revealed the cold-hot nature of CHMs by bioinformatics methods (Liang et al., 2013; Fu et al., 2017). They concluded that cold nature CHMs possessed the tendency to impact cell growth, proliferation, had sedative function, associated with “mental and behavioural disorders” diseases, and hot nature CHMs were related to inflammation and immunity regulation, had cardio-protection function, associated with “endocrine, nutritional and metabolic diseases.” Although scientists have made some achievements in the field of cold-hot nature, the scientific connotation of cold-hot nature needs to be further explained. Our group is committed to revealing the scientific connotation of CHM nature with chemical fingerprint technique.

A number of studies have found that the material basis of the cold-hot nature of CHMs is chemical ingredients (Zhang, 2012; Liang et al., 2013; Wei et al., 2021a). The current research on medicinal nature concentrated on revealing the correlation between cold-hot nature and CHM ingredients. Generally, studies on correlation between cold-hot nature and CHM ingredients mainly included two parts: information representation and nature identification. Information representation was defined as the feature representation of CHM ingredients. Information representation applies chemical fingerprints (Zhang, 2012), molecular descriptors (Liang et al., 2013), metabolomics (Nie et al., 2015) and original effects (Liu et al., 2012) to extract the feature information of CHM ingredients. Chemical fingerprints, including ultraviolet spectrum, infrared spectrum, liquid chromatography, gas chromatography, have been widely used to analyze the ingredient information of CHMs. Molecular descriptors were usually used to extract the information of CHM compounds. Metabolomics applied zoological experiments to extract the ingredient information of CHMs. Original effects mainly included tranquilizing and activating blood, moistening the lung, invigorating the stomach and other attributes. Nature identification introduced machine learning algorithms or built classification algorithms for discriminating cold-hot nature of CHMs. Zhang (2012) utilized chemical fingerprints to analyze CHM ingredients and built nature identification models with machine learning algorithms (such as partial least square method, support vector machine) for nature identification. Our group explored similarity metric models based on chemical fingerprints of CHMs and built nature classification algorithms to classify cold-hot nature of CHMs. Long et al. (2011) and Wang et al. (2016) represented CHM compounds with molecular descriptors, and introduced classical classifiers for discriminating cold-hot nature, respectively. Nie et al. (2015) summarized Metabolomics information of CHMs and built a random forest model to predict the nature of unknown CHMs. Xue’s research group (Liu et al., 2012; Zhang et al., 2012) summarized original efficacy information of CHMs and applied machine learning algorithms for nature identification of CHMs.

As mentioned above, a number of research achievements have been made in the study of cold-hot nature. However, chemical fingerprint technology for nature identification has not been deeply explored. The previous studies focused on the UV thermal chromatogram (Wei et al., 2019b), but less on HPLC. HPLC has high resolution, which can separate complex chemical components to form a chromatogram composed of high and low peaks. Compared with UV spectrum, HPLC can better quantify and characterize the components of CHMs (Qi et al., 2011). It is possible to obtain higher accuracy of cold-hot nature identification by HPLC. Furthermore, most current studies introduced classical machine learning algorithms to establish nature identification models, resulting in low accuracy rates. Designing a special classification algorithm according to the ingredient information of CHMs may obtain higher accuracy rates. In this study, HPLC technology was used to analyze the ingredient information of CHMs. With the obtained HPLC of CHMs, the similarity of CHM ingredients was quantified as a distance metric. Finally, a special nature identification model was constructed to predict the cold-hot nature of CHMs.

## 2 Materials and methods

### 2.1 Chinese herbal medicine dataset

In this study, 61 representative CHMs with clear nature were selected from the classical “Chinese Materia Medica” and “Shen Nong’s Herbal Classic” (Wei et al., 2019a). In these CHMs, 30 CHMs were labeled as cold, and others were labeled as hot. The screening criteria are as follows: 1) Limited to traditional natural plant medicine; 2) CHMs with clear nature, clinically recognizing and no academic dispute. The 61 CHMs are listed in Table 1.

**TABLE 1:** The experimental 61 representative CHMs.

No	Chinese herbal medicines	Nature	Source	Sampling area
1	Platycladi Cacumen	Cold	Mingyi bielu	Linyi, Shandong
2	Kochiae Fructus	Cold	Shen Nong’s Herbal Classic	Feicheng, Shandong
3	Ecliptae Herba	Cold	Tang materia medica	Jinan, Shandong
4	Isatidis Folium	Cold	Mingyi bielu	Tangshan, Hebei
5	Rhei Radix et Rhizoma	Cold	Shen Nong’s Herbal Classic	Dingxi, Gansu
6	Asparagi Radix	Cold	Shen Nong’s Herbal Classic	Huairen, Guizhou
7	Fritillariae Cirrhosae Bulbus	Cold	Shen Nong’s Herbal Classic	Aba, Sichuan
8	Bupleuri Radix	Cold	Shen Nong’s Herbal Classic	Nanyang, Henan
9	Gardeniae Fructus	Cold	Shen Nong’s Herbal Classic	Zhangshu, Jiangxi
10	Rhizoma Anemarrhenae with Peet	Cold	Shen Nong’s Herbal Classic	Baoding, Hebei
11	Sargassum	Cold	Shen Nong’s Herbal Classic	Weihai, Shandong
12	Lophatheri Herba	Cold	Shen Nong’s Herbal Classic	Yuyao, Zhejiang
13	Trichosanthis Fructus	Cold	Shen Nong’s Herbal Classic	Feicheng, Shandong
14	Kansui Radix	Cold	Shen Nong’s Herbal Classic	Shanxi
15	Dried Rehmannia Root	Cold	Shen Nong’s Herbal Classic	Jiaozuo, Henan
16	Dianthi Herba	Cold	Shen Nong’s Herbal Classic	Laiwu, Shandong
17	Fraxini Cortex	Cold	Shen Nong’s Herbal Classic	Lingning
18	Arnebiae Radix	Cold	Shen Nong’s Herbal Classic	Urumqi, Xinjiang
19	Trachelospermi Caulis et Folium	Cold	Shen Nong’s Herbal Classic	Suzhou, Jiangsu
20	Aloe	Cold	Nature theory	Yunnan
21	Puerariae Lobatae Radix	Cold	Shen Nong’s Herbal Classic	Zibo, Shandong
22	Taraxaci_Herba	Cold	Tang materia medica	Linyi, Shandong
23	Menthae Haplocalycis Herba	Cold	Tang materia medica	Haimen, Jiangsu
24	Alizaris Radix	Cold	Tang materia medica	Zhenjiang, Jiangsu
25	Plantaginis Semen	Cold	Shen Nong’s Herbal Classic	Jiujiang, Jiangxi
26	Lonicerae Japonicae Flos	Cold	Tang materia medica	Linyi, Shandong 市
27	Stephaniae Tetrandrae Radix	Cold	Shen Nong’s Herbal Classic	Quzhou, Zhejiang
28	Phellodendri Chinensis Cortex	Cold	Shen Nong’s Herbal Classic	Bazhong, Sichuan
29	Coptidis Rhizome	Cold	Shen Nong’s Herbal Classic	Shizhu, Chongqing
30	Gentianae Radix et Rhizoma	Cold	Shen Nong’s Herbal Classic	Fushun, Liaoning
31	Curculiginis Rhizoma	Hot	Hai Yao Ben Cao	Yibin, Sichuan
32	Pinelliae Rhizoma	Hot	Shen Nong’s Herbal Classic	Dazhou, Sichuan
33	Magnoliae Officinalis Cortex	Hot	Shen Nong’s Herbal Classic	Guangyuan, Sichuan
34	Euodiae Fructus	Hot	Shen Nong’s Herbal Classic	Tongren, Guizhou
35	Arisaematis Rhizoma	Hot	Shen Nong’s Herbal Classic	Heze, Shandong
36	Ephedrae Herba	Hot	Shen Nong’s Herbal Classic	Chifeng, Sichuan
37	Chuanxiong Rhizoma	Hot	Shen Nong’s Herbal Classic	Pengzhou, Sichuan
38	Zingiberis Rhizoma	Hot	Shen Nong’s Herbal Classic	Leshan, Sichuan
39	Corydalis Rhizoma	Hot	Paozhi Lun	Jinhua, Zhejiang
40	Chaenomelis Fructus	Hot	Shen Nong’s Herbal Classic	Xuancheng, Anhui
41	Aucklandiae Radix	Hot	Shen Nong’s Herbal Classic	Lijiang, Yunnan
42	Eucommiae Cortex	Hot	Shen Nong’s Herbal Classic	Mianyang, Sichuan
43	Santali Albi Lignum	Hot	Mingyi bielu	Guangdong
44	Epimedii Folium	Hot	Shen Nong’s Herbal Classic	Shanxi
45	Roasted Corydalis	Hot	Paozhi Lun	Jinhua, Zhejiang
46	Nardostachyos Radix et Rhizoma	Hot	Supplement to Materia Medica	Aba, Sichuan
47	Fructus Piperis Alba	Hot	Tang materia medica	Wenchang, Hainan
48	Mustard Seeds	Hot	Tang materia medica	Anhui
49	Carthami Flos	Hot	Tang materia medica	Xinxiang, Henan
50	Asari Radix et Rhizoma	Hot	Shen Nong’s Herbal Classic	Dandong, Liaoning
51	Notopterygii Rhizoma et Radix	Hot	Shen Nong’s Herbal Classic	Aba, Sichuan
52	Cinnamomi Cortex	Hot	Shen Nong’s Herbal Classic	Hechi, Guangxi
53	Atractylodis Rhizome	Hot	Shen Nong’s Herbal Classic	Jiangsu
54	Alpiniae Katsumadai Semen	Hot	Paozhi Lun	Hainan
55	Piperis Longi Fructus	Hot	Tang materia medica	Wenchang, Hainan
56	Ligustici Rhizoma et Radix	Hot	Shen Nong’s Herbal Classic	Aba, Sichuan
57	Psoraleae Fructus	Hot	Nature theory	Sichuan
58	Aconiti Lateralis Radix Praeparata	Hot	Shen Nong’s Herbal Classic	Jiangyou, Sichuan Province
59	Citri Reticulatae Pericarpium	Hot	Shen Nong’s Herbal Classic	Jiangmen, Guangdong
60	Alpiniae Officinarum Rhizoma	Hot	Mingyi bielu	Zhanjiang, Guangdong Province
61	Clematidis Radix et Rhizoma	Hot	Tang materia medica	Jiangsu

### 2.2 High performance liquid chromatography

In this study, HPLC was applied to represent the ingredient information of CHMs. The experimental methods of HPLC are as follows (Zhang, 2012).

Instruments and materials: Agilent 1100 high performance liquid chromatograph (DAD detector, binary high pressure gradient pump, column temperature box; Agilent); KQ-250E medical ultrasonic cleaner (Qunshan Ultrasonic Instrument Co., Ltd.); Mettler AE240 electronic balance (Mettler, Switzerland). Acetonitrile (Chromatographic pure, American TEDIA company); Wahaha pure water; Other reagents are analytically pure (Tianjin kemio Chemical Reagent Development Center).

Preparation of the test solution is as follows: accurately weigh about 0.5 g of the test medicinal powder (passing 40 mesh), put it in a conical flask with a stopper, accurately add 50 ml of 50% methanol, weigh the mass, and place it in a 60°C water bath for ultrasound extraction for 30 min, after the extraction is completed, let it cool, weigh again to determine the mass, supplement the lost mass with 50% methanol, shake well, filter, and take the continuous filtrate to obtain a 50% methanol extract. Chromatographic conditions is as follows: 1) Chromatographic column: Agilent xdb-c18 column [(4.6 mm) * 250 mm, 5 μm]. 2) Mobile phase: acetonitrile-water (3:97) → acetonitrile-water (100:0), linear gradient elution for 90 min. 3) Flow rate: 1.0 ml/min. 4) Injection volume: 20 ml. 5) Column temperature: 35°C.

The test solution was determined according to the above chromatographic conditions, and the dad (diode array detector) was used for full wavelength scanning of 190–600 nm. Finally, each CHM was collected at 211 wavelengths of 190–400 nm, and the data at 6,524 retention time points were obtained. Because the amount of data was too large for further modeling and analysis, and the chromatographic data of the same CHM at adjacent wavelengths had great correlation, according to the characteristics of UV wavelength, the chromatographic data at representative wavelengths were selected from each CHM. In this work, the chromatographic data at wavelength of 210 nm were applied to study the nature identification model.

### 2.3 High performance liquid chromatography fingerprint similarity

In our previous studies, we proposed a scientific hypothesis that CHMs with similar nature hade similar material basis (Wei et al., 2021a). We have demonstrated this hypothesis with UV spectrum (Wei et al., 2019b; Wei et al., 2021b). In this study, we attempted to demonstrate this hypothesis by building a relationship between CHM ingredients and cold-hot nature. HPLC fingerprints were used to extract the ingredients information of CHMs. Therefore, we explored to reveal that CHMs with similar nature had similar HPLC fingerprints. This means that if the HPLC fingerprints of CHMs are similar, we consider they have the similar medicinal nature.

The similarity of HPLC fingerprints has been widely studied for the quality evaluation of CHMs (Mao, 2020). In this paper, the similarity of HPLC fingerprints was introduced to identify cold-hot nature of CHMs with unknown nature. According to the characteristics of HPLC fingerprints, we defined the similarity of HPLC fingerprints as semantic relevance and fingerprint similarity. Semantic relevance describes the consistency of nature labels, which means that HPLC fingerprints of two CHMs are semantically similar if they have the same labels (cold or hot) (Wei et al., 2018). Fingerprint similarity describes the similarity of ingredient information of CHMs, which means that CHM ingredients related to cold-hot nature are similar. We attempted to learn a Mahalanobis distance to evaluate the similarity of CHM ingredients, which preserved semantic relevance and fingerprint similarity. The smaller the Mahalanobis distance is, the more similar CHM ingredients are.

#### 2.3.1 Distance metric learning

The CHM HPLC dataset is defined as $X=\left[x_{1},\ldots ,x_{n}\right]\in R^{d*n}$ , with $x_{i}\in R^{d}$ being the *i*th CHM HPLC fingerprint in the input space and n being the total number of CHMs, d is the dimension of the fingerprint sample. Denote the Mahalanobis distance between *x*_*i*_ and *x*_*j*_ as (Weinberger et al., 2009):

$$d_{M}(x_{i},x_{j})=\sqrt{{\left(x_{i}-x_{j}\right)}^{T}M(x_{i}-x_{j}})$$ (1)

where superscript ^T^ denotes the transpose of a vector or a matrix, M is a positive semi-definite matrix, which can be decomposed into*M = AA* ^*T*^. Therefore, Eq. 1 can be rewritten as:

$$d_{A}(x_{i},x_{j})=\sqrt{{\left(x_{i}-x_{j}\right)}^{T}AA^{T}(x_{i}-x_{j}})=\left\|A^{T}\left(x_{i}-x_{j}\right)\right\|$$ (2)

According to Eq. 2, learning $d\left(x_{i},x_{j}\right)$ is equal to computing a transformation of Euclidean distance between HPLC fingerprints in the input space. In this study, we learn transformation matrix A according to the similarity of CHM ingredients, including semantic relevance and fingerprint similarity. With the learned matrix A , Mahalanobis distance $d\left(x_{i},x_{j}\right)$ between $x_{i}$ and $x_{j}$ can be calculated by Eq. 2.

#### 2.3.2 Similarity metric

In this study, a Mahalanobis distance was learned to measure the similarity of CHM ingredients. As mentioned above, the similarity of CHM ingredients was quantified as HPLC similarity, including semantic relevance and fingerprint similarity. Most distance metric learning algorithms mainly focused on semantic relevance among CHM fingerprints by learning a distance metric with a given pairwise constraints. Pairwise constraints divide the dataset into two parts, set of equivalence constraints and the set of inequivalence constraints. The set of equivalence constraints is defined as ( $c_{i}$ is the *i*th class):

$$S=\left\{\left(x_{i},x_{j}\right)|x_{i}\in c_{i},\;x_{j}\in c_{i\;}\right\}$$

And the set of inequivalence constraints is defined as ( $c_{i}$ is the *i*th class):

$$D=\left\{\left(x_{i},x_{j}\right)|x_{i}\in c_{i},x_{j}\notin c_{i}\right\}$$

The semantic relevance represents the separability of cold-hot nature, which requires the feature representations of CHM fingerprints in the same class should be closer, and CHM fingerprints in different class should be far away. We built the semantic relevance by optimizing the formula as (Wei et al., 2020):

$$\begin{array}{l} \min\left(\underset{\left(x_{i},x_{j}\right)\in S}{\sum }{\left(y_{i}-y_{j}\right)}^{2}-\lambda \underset{\left(x_{i},x_{j}\right)\in D}{\sum }{\left(y_{i}-y_{j}\right)}^{2}\right)\\ =\min tr\left\{A^{T}\left[\underset{\left(x_{i},x_{j}\right)\in S}{\sum }\left(x_{i}-x_{j}\right){\left(x_{i}-x_{j}\right)}^{T}-\lambda \underset{\left(x_{i},x_{j}\right)\in D}{\sum }\left(x_{i}-x_{j}\right){\left(x_{i}-x_{j}\right)}^{T}\right]A\right\}\\ =\min tr\left(A^{T}PA\right) \end{array}$$ (3)

where λ is a nonnegative tuning parameter, $y_{i}$ is the feature representation of $x_{i}$ by transformation matrix *A*, $P=\underset{\left(x_{i},x_{j}\right)\in S}{\sum }\left(x_{i}-x_{j}\right){\left(x_{i}-x_{j}\right)}^{T}-\lambda \underset{\left(x_{i},x_{j}\right)\in D}{\sum }\left(x_{i}-x_{j}\right){\left(x_{i}-x_{j}\right)}^{T}]$ , the learned matrix *y*_*i*_ is the required transformation matrix.

As mentioned above, fingerprint similarity represents the similarity of CHM HPLC fingerprints, which reflects the similarity of CHM ingredients. Inspired by feature similarity of pulmonary nodule images (Wei et al., 2016), we have built a patch alignment framework for the similarity of gas chromatography. In this study, we introduce this framework for quantifying the similarity of HPLC fingerprints. The patch alignment framework for HPLC fingerprint similarity metric is as follows:

$$\min_{W_{i},b_{i}}\left\|\left(X_{i}^{T}W_{i}+1_{k+1}b_{i}^{T}\right)-Y_{i}{\|}^{2}+\mu \right\|W_{i}{\|}_{F}^{2})$$ (4)

Finally, the global alignment (Wei et al., 2021a) becomes:

$$\min_{Y}tr\left(Y^{T}LY\right)$$ (5)

Given the assumption of linearization that $Y=X^{T}A$ , the global patches errors is calculated as:

$$\underset{A}{mi}ntr\left(A^{T}XLX^{T}A\right)$$ (6)

Therefore, the transformation matrix A in Eq. 3 is learned from semantic relevance, and the transformation matrix A in Eq. 6 is learned from fingerprint similarity. We integrates Eqs 3, 6 to build a similarity metric model. The similarity metric model is as follows:

$$\begin{array}{l} A=argmintr\left(A^{T}\left(P+XLX^{T}\right)A\right)\\ =argmintr\left(A^{T}QA\right) \end{array}$$ (7)

where $Q=P+XLX^{T}$ , the transformation matrix A in Eq. 7 can preserve both semantic relevance and fingerprint similarity.

#### 2.3.3 Projection learning

To solve transformation matrix A in Eq. 7 for a distance metric, it is necessary to avoid redundancy in low dimensional representation of CHM HPLC fingerprints as much as possible. We introduce orthogonal projection learning to solve this problem.

$$\begin{array}{l} A*=argmintr\left(A^{T}QA\right)\\ s.t.\;A^{T}A=I \end{array}$$ (8)

In this case, the optimal projections can be calculated by eigenvalue decomposition on matrix Q , and u eigenvectors of Q corresponding to the u smallest eigenvalues are used to build the optimal solution matrix A* .

### 2.4 Cold-hot nature identification scheme

**FIGURE 1:**
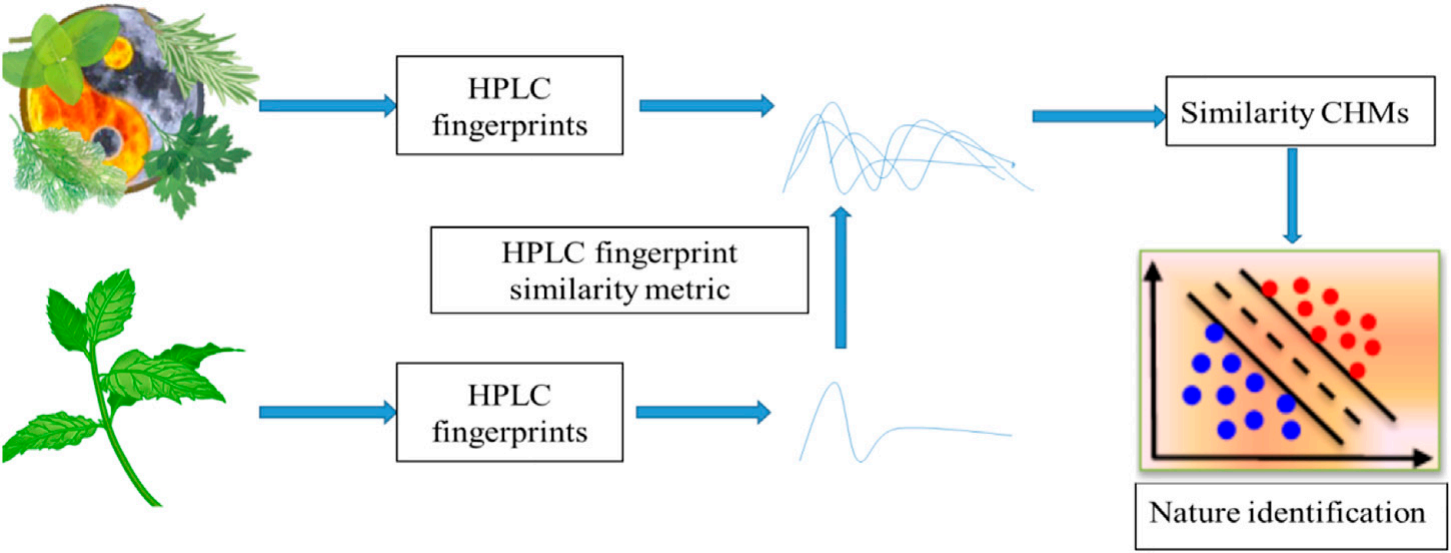
The cold-hot nature identification scheme (CHNIS).

In this study, a cold-hot nature identification scheme (CHNIS) based on similarity metric of HPLC was developed, described in Figure 1. For a CHM with unknown cold-hot nature, we firstly extracted the ingredient information of this CHM by HPLC fingerprints. We then calculated the similarity of HPLC fingerprints between this query CHM and CHMs with clear nature by learning the Mahalanobis distances. The learned Mahalanobis distances were arranged from smallest to largest in order of increasing distance metrics. The most similar r HPLC fingerprints with the smallest distances were selected to search for the most similar r CHMs. Finally, we analyzed the cold-hot nature of this query CHM with the most similar r CHMs. A cold nature probability ( $P_{q}$ ) was computed to analyze the cold degree of the query CHM, which was the ratio of the weight of the cold CHMs to the total weight of CHMs retrieved. The formula is as follows ( c is the number of cold CHMs, h is the number of hot CHMs, r is the number of retrieved CHMs):

$$P_{q}=\frac{\overset{c}{\underset{i=1}{\sum }}W_{i}}{\overset{c}{\underset{i=1}{\sum }}W_{i}+\overset{h}{\underset{j=1}{\sum }}W_{j}},\;c+h=r$$ (9)

where $W_{i}$ is the weight of a CHM, which can be calculated as $W_{i}=1/d_{i}$ , $d_{i}$ is the corresponding Mahalanobis distance. Giving a threshold of $P_{T}$ , if $P_{q}\geq P_{T}$ , we infer that the query CHM is cold, otherwise, it is hot. In this study, we consider $P_{T}$ as 0.5.

### 2.5 The cold-hot nature identification scheme based on similarity metric of High performance liquid chromatography

#### 2.5.1 The cold-hot nature identification algorithm

Given a CHM HPLC dataset $X=\left[x_{1},x_{2},...,x_{n}\right]\in R^{d*n}$ , and the number of nature classes C=2 .

1) Transformation matrix A* establishment. Eigenvalue decomposition on matrix Q to obtain the smallest *u* eigenvectors corresponding to the smallest *u* eigenvalues of Q . Building matrix A* with the smallest *u* eigenvectors.
2) The Mahalanobis distance $d\left(x_{i},x_{j}\right)$ calculation. According to Eq. 2, computing $d\left(x_{i},x_{j}\right)$ between HPLC fingerprints $x_{i}$ and $x_{j}$ with the obtained transformation matrix A* .
3) Similarity metric. Retrieving the *r* most similar CHMs corresponding to the *r* smallest Mahalanobis distances between the query CHM and the CHM dataset.
4) Cold-hot nature classification. Calculating the ratio of the weight of the cold CHMs to the total weight of CHMs retrieved.

### 2.6 Performance evaluation

In this subsection, numerous experiments were built to assess the feasibility of the constructed CHNIS for cold-hot nature classification. We compared the identification performance of our scheme with that of other classical schemes, including retrieval system (RS) (Wei et al., 2019b), Pearson correlation coefficient (PCC) (Wei et al., 2021b), large margin nearest neighbor (LMNN) (Weinberger et al., 2009), information-theoretic metric learning (ITML) (Davis et al., 2007) and extreme learning machine (ELM) (Wei et al., 2021c). RS and PCC were also applied for cold-hot nature classification of CHMs in our studies. LMNN and ITML were classical distance metric learning models. ELM was used for cold-hot-neutral nature prediction of CHM compounds. All experiments of performance assessment were carried out in the environment of CHM HPLC dataset. The application predicted the cold-hot nature of a CHM by studying the similar CHMs with clear nature. We firstly tested the ingredient information of CHMs by HPLC fingerprints. Secondly, we proposed a CHNIS to classify the cold-hot nature of CHMs. Finally, extensive experiments were constructed to verify the feasibility of our proposed scheme.

In the experiments, extrapolation evaluation and stability evaluation were used to test the performance of our CHNIS. Extrapolation evaluation describes the extent to which cold CHMs can be computed based on the retrieved similar CHMs. Extrapolation evaluation divided the CHM dataset into training dataset and test dataset and calculated the probability that the nature of each test CHM belongs to cold. A Receiver Operating Characteristic (ROC) curve was depicted with varying the threshold of the cold probabilities. The area under the ROC curve (AUC) and classification accuracy (ACC) were introduced to evaluate the performance of our proposed CHNIS. The ACC value can be calculated as,

$$ACC=R\left(q_{i}^{r}\right)=\frac{\sum_{j=1}^{r}I\left[y_{i}==y_{j}\right]}{r}$$ (10)

In Eq. 10, $R\left(q_{i}^{r}\right)$ is a function of r , which is the number of retrieved most similar CHMs. $R\left(q_{i}^{r}\right)$ describes a proportion of the accurately predicted CHMs for the i th query CHM in the first r most similar CHMs. The ACC value is the mean of 10 experimental results with randomly selecting the training dataset.

The second evaluation method, stability evaluation, represents the proportion of retrieved CHMs that are semantically relevant to the query CHMs. Leave-one-out method was used to analyze the stability evaluation in the whole CHM HPLC fingerprints. Each time, one CHM was selected as the query sample, and remaining 60 CHMs were used as the training samples. The cold probability of each CHM can be obtained according to the calculated r most similar CHMs in remaining 60 training samples. Therefore, 61 probabilities of CHM dataset were calculated for stability evaluation. Giving a threshold of $P_{T}=0.5$ , we can obtain the calculated label of the 61 CHMs. At last, the AUC and ACC were calculated for evaluating the performance of our scheme.

## 3 Results

### 3.1 Parameter configurations

In our experiments, several parameters in CHNIS were analyzed to classify the cold-hot nature of CHMs. The tuning parameter λ in Eq. 3, parameter μ in Eq. 4 for patch building and the number of retrieved CHMs *r* in CHNIS need to be configured for nature classification. All parameter configurations were studied in the environment of HPLC fingerprints.

**FIGURE 2:**
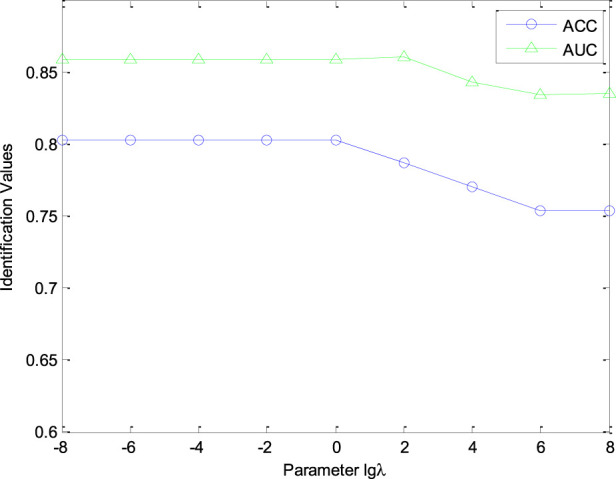
The curves of AUC and ACC value with different λ.

In this study, the stability evaluation was performed to configure the parameters for the optimal CHNIS model. AUC and ACC values were computed to analyze the performance of our CHNIS with varying the parameters ( λ , μ , *r*). Therefore, AUC and ACC were defined as functions of the setting parameters ( λ , μ , *r*) to depict more comprehensive curves for assessing the performance of our CHNIS. We studied the tuning parameter λ in Eq. 3 within the range [10^–8^, 10^–6^, 10^–4^, 10^–2^, 1, 10^2^, 10^4^, 10^6^, 10^8^]. Figure 2 displays the AUC and ACC curves for nature classification of CHM HPLC fingerprints when the tuning parameter λ varies from 10^–8^ to 10^8^. From Figure 2, our CHNIS is more suitable for a smaller parameter λ . When λ≤1 , the prediction performance of our CHNIS is relatively stable. However, the prediction performance decreases with a larger parameter λ . By analyzing ACC and AUC curves, our CHNIS is optimal when defining the parameter λ=1 . In our experiments, parameter μ in Eq. 4 is set as 10^–3^, the number of retrieved CHMs *r* in CHNIS is set as 7.

**FIGURE 3:**
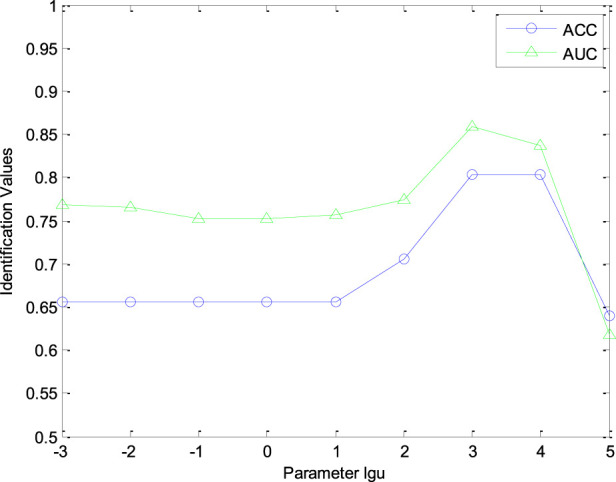
The AUC and ACC curves with different μ.

In this study, we investigated the effect of parameter μ in Eq. 4 for evaluating the performance of cold-hot nature identification. We varied parameter μ within the range [10^–3^, 10^–2^, 10^–1^, 1, 10^1^, 10^2^, 10^3^, 10^4^, 10^5^]. Figure 3 shows the AUC and ACC value curves with different λ for nature prediction. From Figure 3, our CHNIS is sensitive to parameter μ . The AUC curve reaches a peak when $\mu =10^{3}$ . Comprehensively analyzing the AUC and ACC curves, we consider μ as 10^3^. When $\mu =10^{3}$ , our scheme is optimal. The AUC and ACC values of our scheme are 0.8591 and 0.8033, respectively. In this experiment, the parameter λ set as 1, the number of retrieved CHMs *r* is set as 7.

**FIGURE 4:**
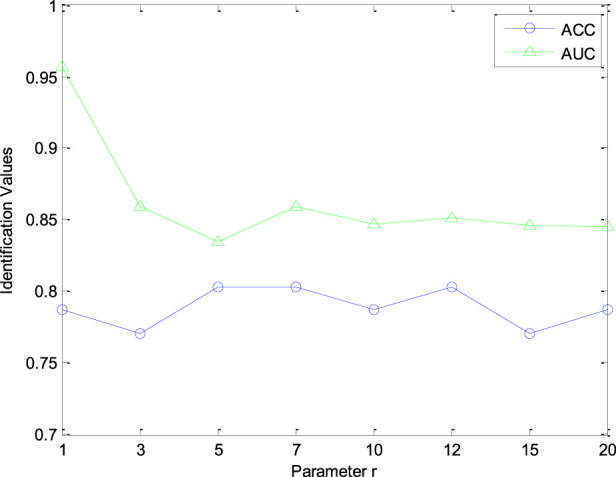
The AUC and ACC curves with different r.

Furthermore, the number of retrieved CHMs *r* in CHNIS was configured for evaluating the identification performance of our model. The value of parameter *r* was test within the range [1, 3, 5, 7, 10, 12, 15, 20]. Figure 4 shows the AUC and ACC curves with different parameter *r*. From this figure, AUC and ACC curves fluctuate with different *r* values, which means that the performance of our CHNIS tends to change slightly with the increase of *r*. Comprehensively analyzing the AUC and ACC curves, our CHNIS achieves optimal performance *r* = 5. In this experiment, the tradeoff parameter λ is set as 1, the parameter μ is set as $10^{3}$ .

### 3.2 Performance evaluation

Several experiments were constructed to demonstrate the feasibility of our proposed CHNIS model for classifying cold-hot nature of CHMs. We compared the classification performance of our CHNIS with that of classifiers applied in our nature studies (RS, PCC, and ELM) and some classical distance metric learning models (i.e., LMNN, ITML). RS and PCC were utilized as comparative references to analyze the similarity of HPLC fingerprints.

Table 2 shows the performance comparison of extrapolation evaluation between CHNIS and other models. Extrapolation evaluation experiments randomly selected 40 CHMs as the training dataset and the remaining CHMs as test dataset. In training dataset, the number of cold CHMs and hot CHMs were about 20, respectively. According to the comparison of identification performance, we draw the following conclusions. Firstly, the identification performance of our scheme CHNIS for cold-hot nature is better than that of the comparison algorithms, which means that CHNIS, comprehensively considering semantic relevance and fingerprint similarity, has the best performance for identifying cold-hot nature. Therefore, our scheme can better mine the ingredient information of CHMs to identify the cold-hot nature. Secondly, distance metric learning algorithms (including ITML, LMNN, RS) are more accurate in nature identification than PCC and ELM. This illustrates that it is more effective for nature identification with similarity metric of CHM ingredients, and also demonstrates the hypothesis that CHMs with similar medicinal nature have similar material basis. Thirdly, ELM with HPLC is poor in predicting cold-hot nature. Finally, the extrapolation evaluation experiments demonstrate the feasibility of our scheme.

**TABLE 2:** Comparison of extrapolation evaluation.

Classifiers	AUC(mean ± std)	ACC(mean ± std)
ITML	0.830 ± 0.047	0.730 ± 0.054
LMNN	0.808 ± 0.032	0.715 ± 0.045
ELM	0.678 ± 0.104	0.671 ± 0.100
RS	0.810 ± 0.050	0.733 ± 0.074
PCC	0.645 ± 0.078	0.548 ± 0.089
**CHNIS**	**0.843 ± 0.036**	**0.800 ± 0.044**

Stability evaluation experiments were preformed to compare the identification performance between CHNIS and other algorithms. Table 3 displays the comparison results, which draws a similar conclusion to Table 2. We reach the following conclusions. Firstly, the performance of our scheme CHNIS outperforms that of the comparison models. Secondly, distance metric learning algorithms used in this study can better mine the ingredients information of CHMs than ELM and PCC in predicting cold-hot nature. Thirdly, our CHNIS has the best stability evaluation. Finally, extrapolation evaluation and stability evaluation experiments comprehensively demonstrate the effectiveness and feasibility of our CHNIS.

**TABLE 3:** Comparison of stability evaluation.

Classifiers	AUC	ACC
ITML	0.815	0.714
LMNN	0.806	0.721
ELM	0.665	0.492
RS	0.831	0.787
PCC	0.642	0.623
**CHNIS**	**0.859**	**0.803**

### 3.3 Nature identification examples

Leave-one-out method was utilized to present the examples of nature identification. Two query CHMs, including Rhei Radix et Rhizoma (cold) and Asari Radix et Rhizoma (hot), were chosen as the instances to interpret the principle of nature identification. Table 4 reports two retrieval CHM instances returned by our CHNIS model. In this table, query CHMs are showed in the second row and top *k* = 7 similar reference CHMs are listed in other rows. The similar reference CHMs were arranged in the order of monotonically increasing Mahalanobis distance. Rhei Radix et Rhizoma was selected as a representative query cold medicine for research. The retrieved CHMs were six reference CHMs with cold nature and one reference CHM with hot nature. The cold nature probability was calculated as 92.1%, which meant that the query CHMs was probably cold. Asari Radix and Rhizoma was selected as a representative query hot medicine for research. The retrieved CHMs were six reference CHMs with hot nature and one reference CHM with cold nature. The cold nature probability was calculated as 6.9%, which meant that the query CHMs was probably hot. The identification examples indicate that there is a correlation between CHM ingredients and cold-hot nature.

**TABLE 4:** The nature identification examples. The top *k* = 7 similar CHMs are arranged in the order of monotonically increasing Mahalanobis distance. Cold/hot nature labels are denoted in the brackets.

Prediction examples	CHMs with cold nature	CHMs with hot nature
Query CHMs	Rhei Radix et Rhizoma (cold)	Asari Radix et Rhizoma (hot)
The similar reference CHMs	Puerariae Lobatae Radix (cold)	Aconiti Lateralis Radix Praeparata (hot)
	Stephaniae Tetrandrae Radix (cold)	Ligustici Rhizoma et Radix (hot)
	Phellodendri Chinensis Cortex (cold)	Clematidis Radix Et Rhizoma (hot)
	Psoraleae Fructus (hot)	Arisaematis Rhizoma (hot)
	Plantaginis Semen (cold)	Corydalis Rhizoma (hot)
	Sargassum (cold)	Mustard Seeds (hot)
	Isatidis Folium (hot)	Trachelospermi Caulis et Folium (cold)

### 3.4 Overall identification performance

In this study, the overall performance of our CHNIS was assessed with evaluation indices, including confusion matrix, recall, precision and F-score. All evaluation indices were calculated by leave-one-out method. The confusion matrix for nature identification of 61 CHMs is shows in Table 5. The prediction accuracy rate of cold CHMs is 83.3% (25/30), while the classification accuracy rate of hot CHMs is 77.4% (24/31). Therefore, the total identification accuracy rate of 61 CHMs is 80.3% (49/61). Our scheme has higher prediction accuracy for cold CHMs, but lower prediction accuracy for hot CHMs. The recall, precision and F-score of nature prediction of 61 CHMs are showed in Table 6. From Tables 5, 6, we find that our scheme is effective in the prediction of cold-hot nature with HPLC fingerprints. CHM ingredients are closely related to cold-hot nature.

**TABLE 5:** Confusion matrix of 61 CHMs.

Ground truth	Identification	
	Cold	Hot
Cold	25	5
Hot	7	24

**TABLE 6:** The recall, precision and F-score of 61 CHMs.

	Cold (%)	Hot (%)
Recall	83.3	77.4
Precision	78.1	82.8
F-score	80.6	80.0

## 4 Discussion

HPLC is an important analytical method in the research of CHM ingredients in recent years. HPLC can quantitatively and qualitatively analyze the ingredients of CHMs. Our group previously used UV spectrum to analyze the ingredients of CHMs, and established a nature identification model for classifying cold-hot nature. Table 7 displays the performance comparison of cold-hot nature evaluation of related studies. All related studies are carried out in the environment of 61 representative CHM dataset. The experimental results show that HPLC has a higher prediction accuracy rate, which means that HPLC can better extract the characteristics of cold-hot nature of CHM ingredients.

**TABLE 7:** Performance comparison of cold-hot nature evaluation.

Related study	ACC
Zhang et al. (2012)	0.632
RS Wei et al. (2019b)	0.787
PCC Wei et al. (2021b)	0.623
MSSMRS Wei et al. (2019a)	0.705
**CHNIS**	**0.803**

HPLC fingerprints present a challenge to classical classification algorithms, which leads to a low classification accuracy. The same conclusion is also obtained in the traditional similarity metric methods, such as Pearson correlation. These methods perhaps suffer from high dimensionality of data. Furthermore, the classical algorithms are all general classification algorithms. These methods may not be able to mine the characteristic information of CHM ingredients, which makes the identification accuracy of cold-hot nature low.

According to the hypothesis that CHMs with the same cold-hot nature have the similar material basis, a distance metric learning method is introduced to quantify the similarity of CHM ingredients as distance metric, and build a cold-hot nature identification scheme for understanding material basis of CHMs. Distance metric learning methods, such as LMNN and ITML, only focus on the semantic of relevance of CHM ingredients without considering fingerprint similarity. Experiments indicate that semantic relevance alone cannot represent all the similarity measures. Here we combine semantic relevance and fingerprint similarity to represent the similarity of CHM ingredients. This model can better mine the information of CHM ingredients. Experiments find that fingerprint similarity can improve the performance of the model. We assume fingerprint similarity is an important part of similarity measurement.

As a classical classifier, ELM used in our study is a general classifier, which does not consider the characteristics of CHM fingerprints. This results in low nature prediction accuracy because of the small samples and high dimensionality of CHM fingerprints in this study. Our CHNIS is built for cold-hot nature identification base on the hypothesis that CHMs with the same cold-hot nature have similar material basis. Compared with classical classifiers, our CHNIS not only models the class separability of fingerprints, but considers the fingerprint similarity. The experimental results also show that our model achieves good classification rate.

However, there are some limitations to our study. First, this research only analyzes CHM ingredients with HPLC fingerprints. Other chemical fingerprints are not taken into account in this study. Multi-fingerprints technology perhaps improves the nature identification accuracy. Therefore, multi-fingerprints fusion algorithm for cold-hot nature identification of CHMs is the focus of follow-up attention. Second, we define the similarity of CHM HPLC as a distance metric. The HPLC fingerprints are small sample and high dimension, which makes classifiers difficult to perform. On the basis of such characteristics, we are committed to designing the forecasting models in the future. Third, this study focuses on building a similar model to identify cold-hot nature of CHMs by CHM HPLC characteristics. The information of CHM ingredients has not been thoroughly mined. In the future, we will explore other chemical fingerprints to extract the information of CHM ingredients for cold-hot nature classification.

## 5 Conclusion

In this study, a CHNIS for cold-hot nature identification of CHMs is proposed. CHM HPLC fingerprints are applied to extract the information of CHM ingredients. Based on CHM HPLC fingerprints, effective experiments demonstrate that the performance of our scheme outperforms that of the comparative classifiers in classifying cold-hot nature. The overall identification accuracy of 61 CHMs reached 80.3%. According to the experimental results, we find that CHM ingredients are closely related to the cold-hot nature of CHMs. Furthermore, we demonstrate the feasibility of scientific hypothesis that CHMs with the same cold-hot nature have similar material basis.

## Data Availability

The raw data supporting the conclusion of this article will be made available by the authors, without undue reservation.
